# ChIP-seq and ChIP-exo profiling of Pol II, H2A.Z, and H3K4me3 in human K562 cells

**DOI:** 10.1038/sdata.2018.30

**Published:** 2018-03-06

**Authors:** Zenab F. Mchaourab, Andrea A. Perreault, Bryan J. Venters

**Affiliations:** 1 Department of Molecular Physiology and Biophysics, Vanderbilt Genetics Institute, Vanderbilt Ingram Cancer Center, Vanderbilt University, Nashville, TN 37232, USA; 2 Chemical and Physical Biology Program at Vanderbilt University, Nashville, TN 37232, USA

**Keywords:** Histone variants, Histone post-translational modifications, Transcriptomics

## Abstract

The human K562 chronic myeloid leukemia cell line has long served as an experimental paradigm for functional genomic studies. To systematically and functionally annotate the human genome, the ENCODE consortium generated hundreds of functional genomic data sets, such as chromatin immunoprecipitation coupled to sequencing (ChIP-seq). While ChIP-seq analyses have provided tremendous insights into gene regulation, spatiotemporal insights were limited by a resolution of several hundred base pairs. ChIP-exonuclease (ChIP-exo) is a refined version of ChIP-seq that overcomes this limitation by providing higher precision mapping of protein-DNA interactions. To study the interplay of transcription initiation and chromatin, we profiled the genome-wide locations for RNA polymerase II (Pol II), the histone variant H2A.Z, and the histone modification H3K4me3 using ChIP-seq and ChIP-exo. In this Data Descriptor, we present detailed information on parallel experimental design, data generation, quality control analysis, and data validation. We discuss how these data lay the foundation for future analysis to understand the relationship between the occupancy of Pol II and nucleosome positions at near base pair resolution.

## Background & Summary

Control of eukaryotic transcription patterns involves the interplay of RNA polymerase II (Pol II) and chromatin. In metazoans, once Pol II initiates transcription, it rapidly transitions to a regulated paused state, 30-50 base pairs (bp) downstream of the transcription start site (TSS)^1^. In this position, Pol II is juxtaposed with the first nucleosome downstream of the TSS^2,3^. The +1 nucleosome is specifically enriched with the histone variant H2A.Z and tri-methylation of the fourth N-terminal lysine on the histone H3 tail (H3K4me3). It has been known for several decades that Pol II must overcome nucleosomal obstacles during transcription^4^. However, questions remain regarding the molecular mechanisms underlying how chromatin regulates Pol II activity, and vice versa.

Since functional genomic approaches often require tens of millions of cells per assay, immortalized mammalian cell lines are frequently used in these studies. Due to its facile growth characteristics and its designation as an ENCODE tier 1 cell line, K562 cells are one of the most commonly used mammalian cell lines. The K562 cell line was originally established from a female patient with chronic myeloid leukemia^5^. K562 cells are considered erythroleukemic, displaying characteristics of undifferentiated granulocytes and erythrocytes^6^. In the presence of specific chemical inducers, K562 cells will differentiate along the erythroid lineage and upregulate globin expression^7–9^.

As functional genomic technologies improve, they present new opportunities to address key biological questions. Chromatin immunoprecipitation coupled to high throughput sequencing (ChIP-seq) is a powerful tool to study mechanisms of gene regulation by selectively enriching for DNA fragments that interact with a given protein in living cells. Briefly, in vivo protein-DNA interactions are preserved through covalent linkage as a result of formaldehyde treatment. Cells are lysed, the nuclear fraction is retained, and the chromatin is fragmented by sonication to 100–500 bp. DNA fragments interacting with the protein of interest are enriched by ChIP, and a library is prepared by adding sequencing adapters according to manufacturer’s instructions. Genomic regions that interact with the protein of interest are deduced by sequencing from the sonication borders, which are typically several hundreds of base pairs away from the protein-DNA crosslinked interaction site.

A more recently developed technology, called ChIP-exo, improves upon ChIP-seq by providing near base pair mapping resolution for protein-DNA interactions. The key innovation of the ChIP-exo methodology is the incorporation of lambda exonuclease digestion in the library preparation workflow to effectively footprint the left and right 5′ DNA borders of the protein-DNA crosslink site. Thus, rather than sequencing from the distal sonication borders as in ChIP-seq, ChIP-exo enriched DNA fragments are sequenced from the left and right 5′ DNA borders of the protein-DNA crosslink site. The precision of the resulting data can be leveraged to provide unique and ultra-high resolution insights into the functional organization of the genome. Given its high base pair resolution, ChIP-exo is uniquely capable of spatially resolving divergent, initiating, paused, and elongating RNA polymerase II on a genome-wide scale. For example, our related work used Pol II ChIP-exo analysis in K562 cells to show that divergent transcription at promoters arises from distinct, resolvable pre-initiation complexes (PICs)^10^. Reanalysis of this data by Lis and colleagues showed that enhancers and promoters share a unified transcription initiation architecture^11^. Sandelin and colleagues repurposed our Pol II ChIP-exo data to provide corroborative evidence for alternative transcription initiation within closely spaced promoters^12^. Finally, reanalysis by Lukatsky and colleagues found a DNA triplicate code linked to PIC positioning at promoters^13^.

**Figure 1:**
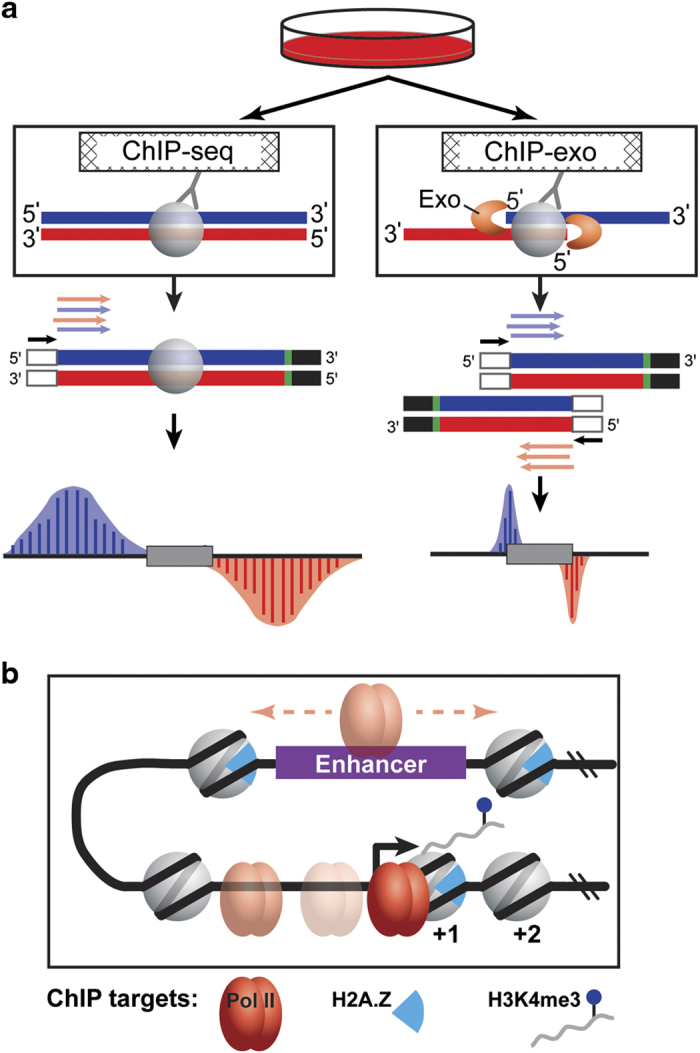
Experimental design and overview of ChIP targets. (**a**) K562 cells were cultured using standard conditions and harvested for ChIP-seq and ChIP-exo. ChIP-seq reports on the sonication borders of ChIP-enriched DNA fragments, wherein the location of the protein-DNA crosslink is deduced. In contrast, ChIP-exo sequences the exonuclease left and right borders that flank protein-DNA interactions. (**b**) Illustration of biological context of ChIP targets: Pol II, H2A.Z, and H3K4me3.

**Figure 2:**
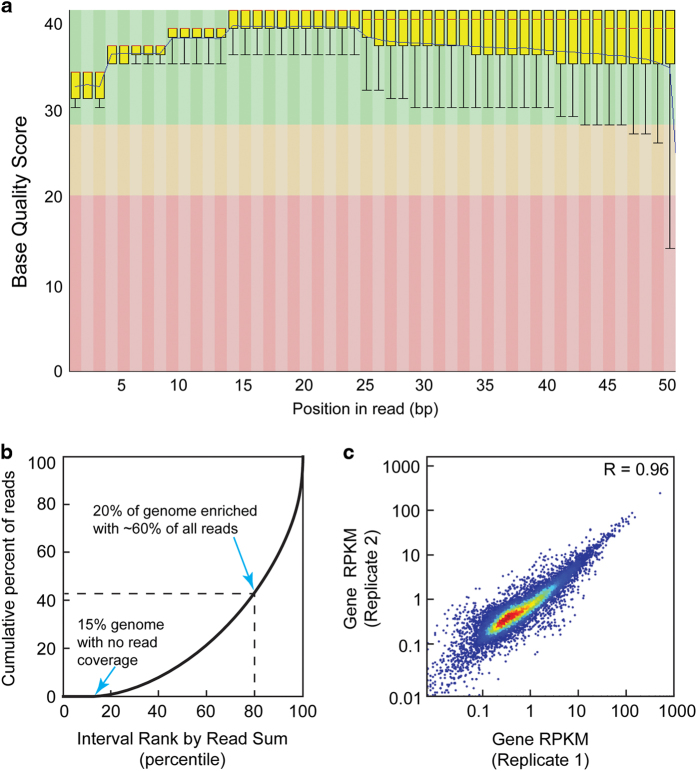
Quality control, enrichment analysis, and reproducibility for ChIP-seq and ChIP-exo data. (**a**) Box-plot distribution of base quality scores are shown for Pol II ChIP-exo replicate 1. A score greater than 30 (green region) indicates a high confidence base call. (**b**) ChIP-enrichment analysis plot that displays the cumulative percent of total reads found in a given percent of the mappable human genome. No ChIP enrichment would result in a diagonal trace. (**c**) Scatter plot correlation analysis for Pol II ChIP-exo biological replicates as measured by the Spearman correlation coefficient R-values (upper right corner).

**Figure 3:**
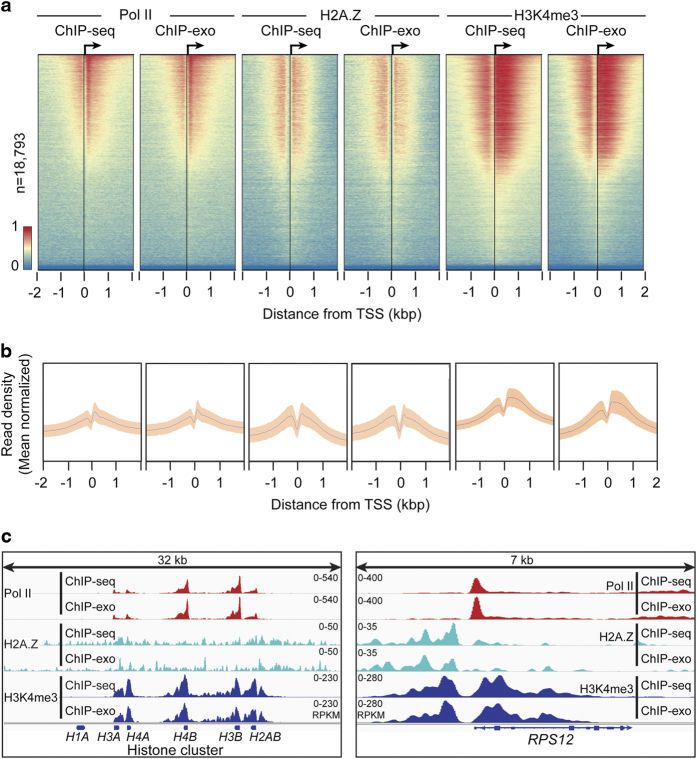
Genomic distribution of Pol II, H2A.Z, and H3K4me3. (**a**) Row-linked heatmaps show RPKM normalized number of reads across a 4 kb genomic interval in 20 bp bins relative to the TSS. Heatmaps were generated from merged biological replicate pairs for Pol II, H2A.Z, and H3K4me3. Regions are sorted in descending order based on average row tag density for Pol II ChIP-seq. Each row represents a gene, with 18,793 genes displayed. Red and blue reflect high and low read densities, respectively. (**b**) Composite plots below each heatmap quantify the normalized tag density. The central trace denotes the average tag density for each 20 bp bin and the orange fill reflects the standard deviation. (**c**) Genome browser view of ChIP-seq and ChIP-exo signal for Pol II, H2A.Z, and H3K4me3 in K562 cells shown at a histone cluster locus and the *RPS12* gene. Tag distributions were smoothed and RPKM normalized using deepTOOLS. Traces were generated from merged biological replicate pairs for Pol II, H2A.Z, and H3K4me3.

In this Data Descriptor, we extend the value of our previous Pol II ChIP-exo data by generating 12 new ChIP-seq and ChIP-exo data sets for Pol II, H2A.Z, and H3K4me3 in K562 cells. ChIP-exo mapping of Pol II, a histone variant, and a histone modification should enable other investigators to use these data sets for their own research to further understand the detailed interplay of Pol II and chromatin. Further, paired libraries generated side-by-side should enable direct comparisons between the quality of ChIP-seq and ChIP-exo mapping genome-wide. On average, 42 million uniquely aligned reads were generated for each ChIP-seq and ChIP-exo data set (Table 1). To facilitate interpretation of these data, we provide detailed information on experimental design (Fig. 1), sequence quality control analyses (Fig. 2), and biological validation (Fig. 3).

## Methods

### Tissue culture

Human chronic myelogenous leukemia cells (K562, ATCC) were maintained at 37 ^o^C in 5% CO_2_ between 0.1–1 million cells/ml in DMEM (Dulbecco’s Modified Eagle Media) containing 10% bovine calf serum and 1% Penicillin/Streptomycin.

### ChIP-seq and ChIP-exo library preparation

ChIP-exo was performed as previously described^10,14^ with chromatin extracted from 50 million cells, ProteinG MagSepharose resin (GE Healthcare), and 5 ug of antibody directed against RNA polymerase II, H2A.Z, or H3K4me3, (Santa Cruz sc899, EMD Millipore 07-594, or Abcam ab8580, respectively). For each biological replicate, ChIP-seq and ChIP-exo libraries were prepared using the same starting sonicated nuclear extract. Importantly, this controls for more direct comparisons ChIP-seq and ChIP-exo for each antibody used. Libraries were sequenced using an Illumina NextSeq500 sequencer as single-end reads 50 or 75 nucleotides in length (Table 1).

### Sequence read alignment and quality control

The base call quality for each sequenced read was assessed using the FastQC program (bioinformatics.babraham.ac.uk/projects/fastqc/) (Fig. 2a and Supplementary Figs. 1–2). Sequence reads (fastq files) were aligned to the human hg19 reference genome build using BWA-MEM algorithm with default parameters^15^. The resulting bam files were first sorted using the Samtools Sort function, and then bam index files were generated using the Samtools Index function^16^. The purpose of bam index files is to enable viewing of raw sequencing data in a genome browser. Next, genome-wide read coverage and enrichment were assesses using deepTOOLS fingerprint plots^17^ (Fig. 2b and Supplementary Fig. 3).

### Biological validation

To estimate variance across biological replicates, the Pearson correlation coefficient for pairwise gene Reads Per Kilobase of genome per Million reads (RPKM) was computed (Fig. 2c, Supplementary Fig. 4) using the HOMER suite (Hypergeometric Optimization of Motif EnRichment)^18^. Briefly, bam files were converted to tag directories using the makeTagDirectory function with the –genome, –checkGC, and –format options. To quantify and normalize tags within gene body regions to RPKM, the analyzeRepeats function was used with the –rpkm and –d options (Data Citation 1).

ChAsE (Chromatin Analysis and Exploration) visualization suite^19^ was used to display the distribution of Pol II, H2A.Z, and H3K4me3 relative to the TSS (Fig. 3a and b, Supplementary Fig. 6). Raw sequencing tags were binned, smoothed, and RPKM computed using the deepTOOLS genomeCoverage tool (20 bp bin, 100 bp sliding window)^17^. Smoothed RPKM signal was visualized with Integrative Genomics Viewer (IGV) (Fig. 3c)^20^.

### Code availability

Below is a list of software used in this study.

^15^BWA-MEM v0.7.13

^16^Samtools v1.3.1

FastQC v0.11.2 (bioinformatics.babraham.ac.uk/projects/fastqc/)

^17^deepTOOLS v2.2.4

^21^bedTOOLS v2.24.0

^18^HOMER v4.6

^20^IGV v2.3.77

^19^ChAsE v1.0.11

### Data Records

ChIP-seq and ChIP-exo bigwig data files were deposited in the NCBI Gene Expression Omnibus (GEO) under accession number GSE108323 (Data Citation 2). GEO linked ChIP-seq and ChIP-exo bam data files were deposited in the Sequence Read Archives (SRA) under accession number SRP116017 (Data Citation 3). SAMN07546015 (Table 1) contains data of biological replicate 1 whose reads were 50 nucleotides in length (except for H3K4me3 ChIP-seq replicate 1, which has 75 nucleotide reads) and SAMN07546016 (Table 1) contains data of biological replicate 2 whose reads were 75 nucleotides in length.

### Technical Validation

#### Overview of experimental design

In this study, functional genomic experiments using K562 cells were designed with two primary goals in mind. First, to facilitate direct comparisons for each biological replicate, ChIP-seq and ChIP-exo were performed on pooled fractions of sonicated nuclear extracts. Second, the ChIP targets (Pol II, H2A.Z, and H3K4me3) were selected so that the spatial relationships between Pol II and nucleosome positions may be examined on a genome-scale at high precision (Fig. 1a, b). H2A.Z and H3K4me3 are associated with both proximal promoters and distal enhancers. Indeed, recent reports have underscored the interplay of these proteins in Pol II recruitment, enhancer RNA transcription, and enhancer-promoter interactions^22–24^. Taken together, reanalysis of this collection of data should enable new biological insights into chromatin dynamics during transcriptional activation. Below, we briefly describe the rationale and considerations for sequencing data analysis with respect to general read quality, genome alignment, ChIP enrichment, replicate correlation, and biological validation.

#### Raw sequence quality control analyses

To assess the quality of the raw sequencing data sets, base call scores were analyzed using the FastQC program and displayed as a box plot distribution at each base position (Fig. 2a and Supplementary Figs. 1–2). The average base quality score for all 12 ChIP data sets in the present study fell within the high confidence range (base quality score of 30–40, green region).

Raw sequence reads were aligned to the hg19 build of the human genome. On average, 46 million total aligned reads were generated for each ChIP-seq and ChIP-exo data set (Table 1), ranging from 20-95 million reads. Because of the ambiguity of reads that align to multiple locations throughout the genome, we only retain uniquely aligned reads for subsequent analyses. On average, 42 million uniquely aligned reads were obtained per data set, representing unique alignment rates between 84-93%.

Two critical questions for assessing ChIP sequencing data quality are: 1) how much of the genome is represented by a given experiment? and 2) to what extent did the ChIP assay enrich for specific regions of the genome? Typically, high genome coverage and strong ChIP enrichment are desirable in ChIP experiments. To determine genome coverage and ChIP enrichment simultaneously, we used the deepTOOLS suite to perform a fingerprint analysis (Fig. 2b). In the case of Pol II ChIP-exo (Fig. 2b), the fingerprint plot trace intersects the x-axis at 15, indicating 85% genome coverage. In fingerprint plots, a rightward deflection of the trace indicates the extent of ChIP enrichment. Given a point along the trace that is the point of intersection from the axes, the corresponding values on the x- and y-axes denote the percent of genome and the percent of all uniquely aligned reads, respectively. Together, these values reflect ChIP enrichment.

For example, the Pol II ChIP-exo fingerprint trace reveals that 20% of the genome (x-axis, 100-80) is enriched with 60% of all uniquely aligned reads (y-axis, 100-40), suggesting strong enrichment Pol II ChIP-exo data. Fingerprint plots for other replicates showed similar patterns of genome coverage and ChIP enrichment (Supplementary Fig. 3). Theoretically, complete genome coverage with no enrichment would be result in a trace with a slope equal to one that intersects the origin (eg: whole genome sequencing wherein 50% of the genome is contains 50% of all aligned reads).

#### Biological validation

After verifying the quality of the raw sequencing data, we next sought to provide evidence of biological validity for the data. First, we determined the extent to which biological replicates were reproducible using correlation scatter plots (Fig. 2c). For each gene, the RPKM was computed using the HOMER suite (Data Citation 1). Pearson correlation coefficients (R-values) were computed for pairwise correlation plots of gene RPKMs across biological replicates. For example, biological replicates for Pol II ChIP-exo analysis displayed an R-value of 0.96, indicating high reproducibility (Fig. 2c). Correlation analysis of other data resulted in positive R-values between 0.56 and 0.99 (Supplementary Fig. 4). Similarity across ChIP-seq and ChIP-exo for each factor were assessed by correlation analysis between merged ChIP-exo and ChIP-seq data sets, which displayed R-values between 0.86 and 0.99 (Supplementary Fig. 5).

Given that certain transcription factors operate at a consistent distance from TSSs, analyzing global patterns of ChIP signal relative to TSSs is a useful method to assess biological validation. It is well established that once Pol II initiates transcription of genes in metazoans, Pol II moves into a stable paused state 30–50 bp downstream of the TSS^1^. Likewise, H2A.Z and H3K4me3 are consistently incorporated primarily into the +1 nucleosome of actively transcribed genes^25^. Thus, to examine global patterns of ChIP enrichment, the Chromatin Analysis and Exploration (ChAsE) heatmap tool was used to align ChIP signal merged from both biological replicates to TSSs (Fig. 3a, sorted by max peak; and Supplementary. Fig. 6, sorted by max peak position). Quantification of signal density relative to TSSs is displayed as a composite plot below each heatmap (Fig. 3b). As expected, Pol II ChIP signal was sharply enriched just downstream of the TSS at the pause site for both ChIP-seq and ChIP-exo data. H2A.Z and H3K4me3 signals were broadly enriched up- and downstream of the TSS, consistent with the −1 and +1 nucleosome positions. To examine individual examples of global patterns, RPKM normalized tracks for ChIP signal were displayed using the Integrative Genome Viewer (IGV). The distribution of ChIP signal at a histone cluster and the *RPS12* gene recapitulated the global patterns of Pol II, H2A.Z, and H3K4me4 (Fig. 3c). Taken together, we conclude that the data presented in this Data Descriptor represent high quality next generation sequencing data that are biologically valid, and should be useful to future studies that seek to understand the interplay of Pol II and chromatin in high resolution on a global scale.

## Additional information

**How to cite this article**: Mchaourab, Z. F. *et al.* ChIP-seq and ChIP-exo profiling of Pol II, H2A.Z, and H3K4me3 in human K562 cells. *Sci. Data* 5:180030 doi: 10.1038/sdata.2018.30 (2018).

**Publisher’s note:** Springer Nature remains neutral with regard to jurisdictional claims in published maps and institutional affiliations.

## Supplementary Material



Supplementary Information

## Figures and Tables

**Table 1:** Sequencing read alignment statistics for ChIP-seq and ChIP-exo.

**ChIP target**	**Antibody**	**Assay**	**Replicate**	**Sample ID**	**Total Mapped Reads**	**Uniquely Mapped Reads**	**Unique Mapping Rate**
**Pol2**	sc899 (Santa Cruz)	ChIP-seq	1	SAMN07546015	28,247,807	24,603,174	87%
		ChIP-seq	2	SAMN07546016	94,976,221	85,443,543	90%
		**TOTAL**			**123,224,028**	**110,046,717**	
		ChIP-exo	1	SAMN07546015	38,286,067	32,989,473	86%
		ChIP-exo	2	SAMN07546016	56,147,982	50,109,500	89%
		**TOTAL**			**94,434,049**	**83,098,973**	
**H2A.Z**	07-594 (EMD Milipore)	ChIP-seq	1	SAMN07546015	23,004,351	20,145,444	88%
		ChIP-seq	2	SAMN07546016	35,928,019	32,559,626	91%
		**TOTAL**			**58,932,370**	**52,705,070**	
		ChIP-exo	1	SAMN07546015	27,043,543	23,726,092	88%
		ChIP-exo	2	SAMN07546016	64,925,557	58,936,047	91%
		**TOTAL**			**91,969,100**	**82,662,139**	
**H3K4me3**	ab8580 (Abcam)	ChIP-seq	1	SAMN07546015	57,472,144	52,954,309	92%
		ChIP-seq	2	SAMN07546016	56,431,834	52,528,142	93%
		**TOTAL**			**113,903,978**	**105,482,451**	
		ChIP-exo	1	SAMN07546015	19,935,227	16,778,098	84%
		ChIP-exo	2	SAMN07546016	52,325,622	47,444,745	91%
		**TOTAL**			**72,260,849**	**64,222,843**	
