# Supplementary material for: ChIP-seq and ChIP-exo profiling of Pol II, H2A.Z, and H3K4me3 in human K562 cells
Source: Sci Data. 2018 Mar 6;5:180030. doi: 10.1038/sdata.2018.30 (PMC5839155; doi:10.1038/sdata.2018.30)
Supplement: Supplementary Information [file sdata201830-s2.pdf]

Supplementary Figures for:

Zenab F. Mchaourab, Andrea A. Perreault, and Bryan J. Venters. ChIP-seq and ChIP-exo profiling of Pol II, H2A.Z, and H3K4me3 in human K562 cells. *Nature Scientific Data*. (2018)

Table of Contents

**Figure title**	**Page number**
1. FastQC base quality analysis for biological replicate 1 data sets	2
2. FastQC base quality analysis for biological replicate 2 data sets	3
3. Fingerprint plots to assess genome coverage and ChIP enrichment for ChIP-seq and ChIP-exo from merged biological replicates	4
4. Scatter plot correlation analysis for Pol II ChIP-exo biological replicates as measured by the Spearman correlation coefficient R-values (upper left corner)	5
5. Scatter plot correlation analysis for ChIP-exo and ChIP-seq libraries as measured by the Spearman correlation coefficient R-values (upper left corner)	6
6. ChAsE heatmap display for Pol II, H2A.Z, and H3K4me3 ChIP signal from merged biological replicates	7

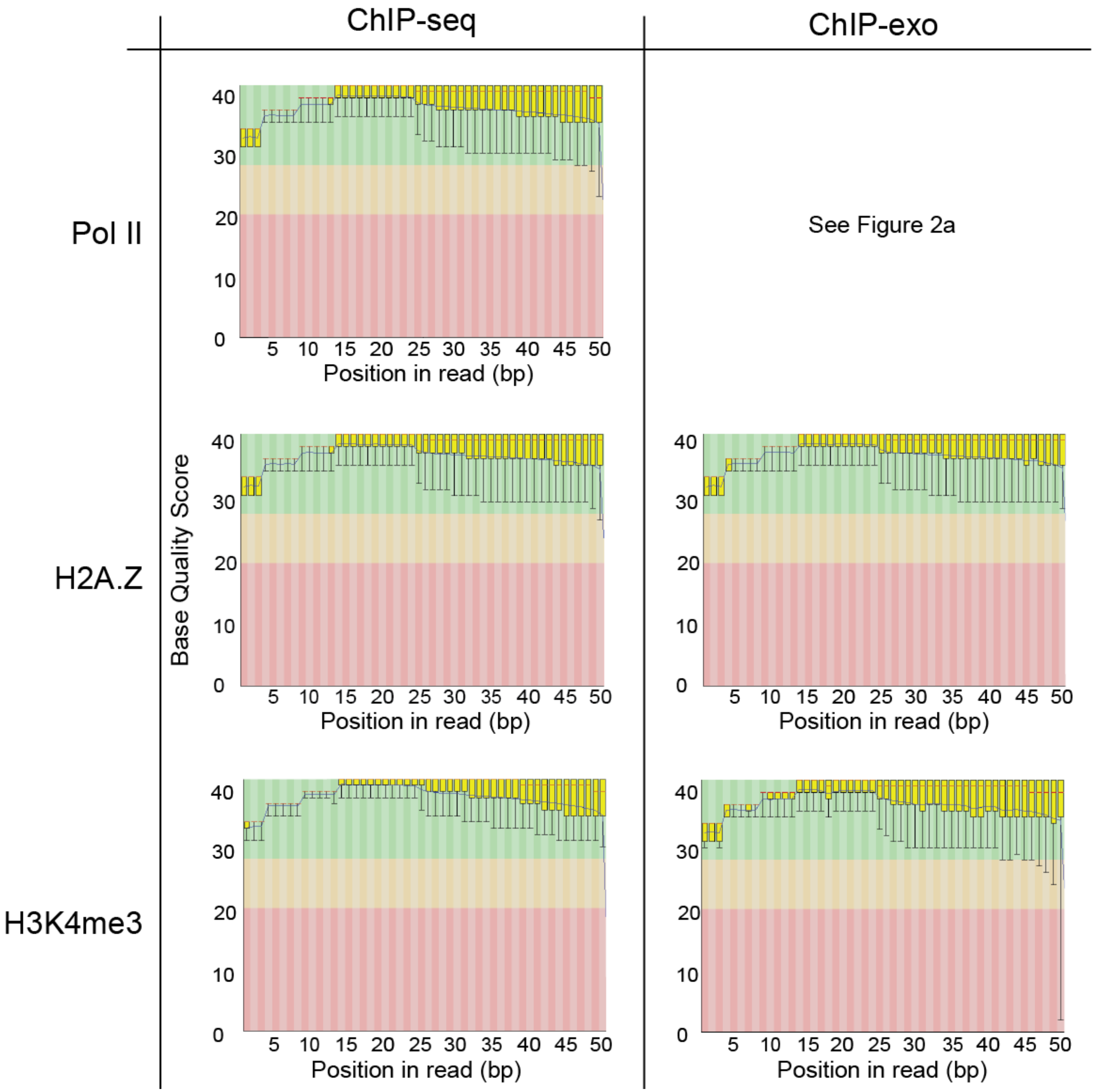

Supp. Fig. 1. FastQC base quality analysis for biological replicate 1 data sets. Related to Fig. 2a.

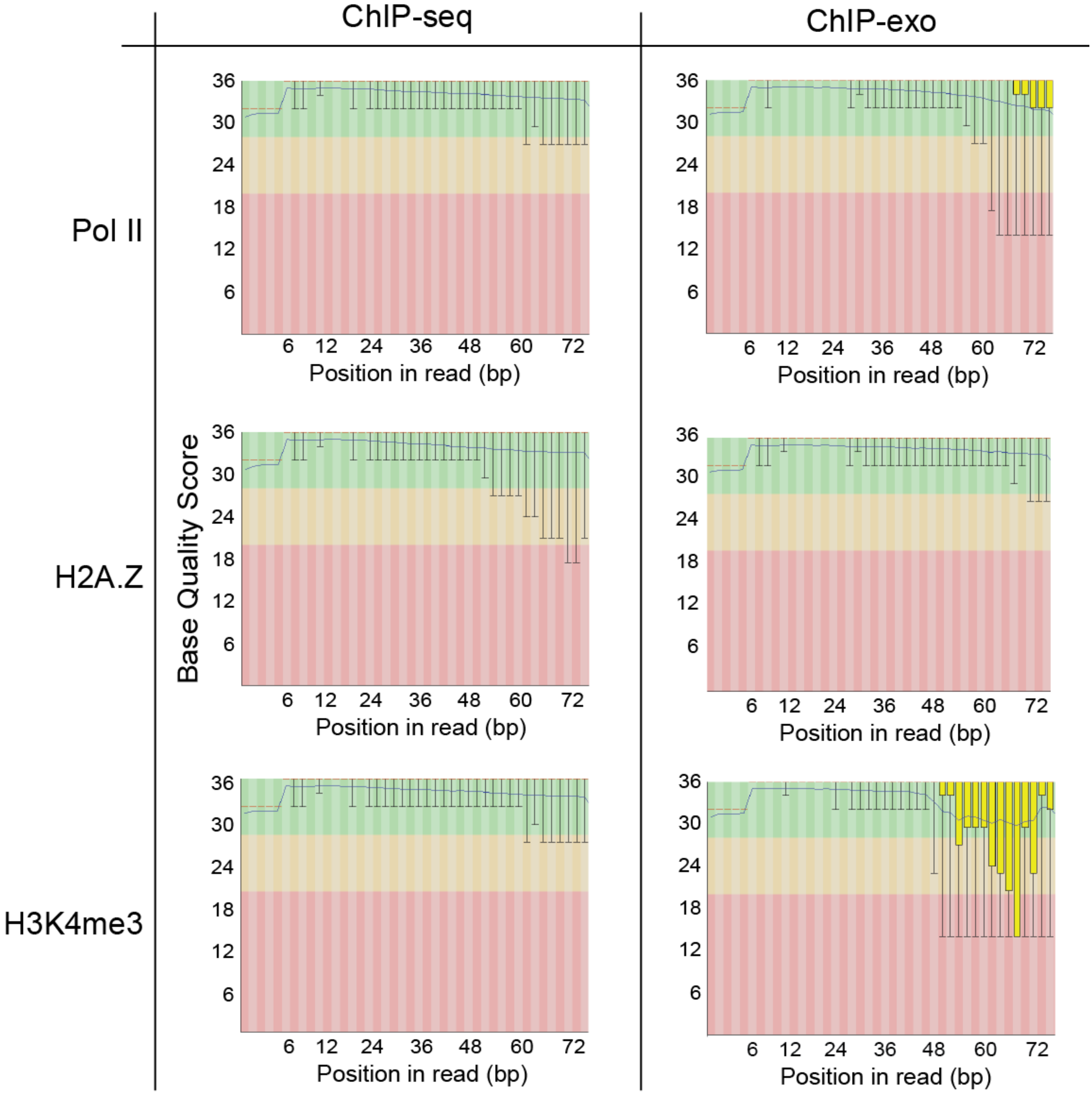

Supp. Fig. 2. FastQC base quality analysis for biological replicate 2 data sets. Related to Fig. 2a.

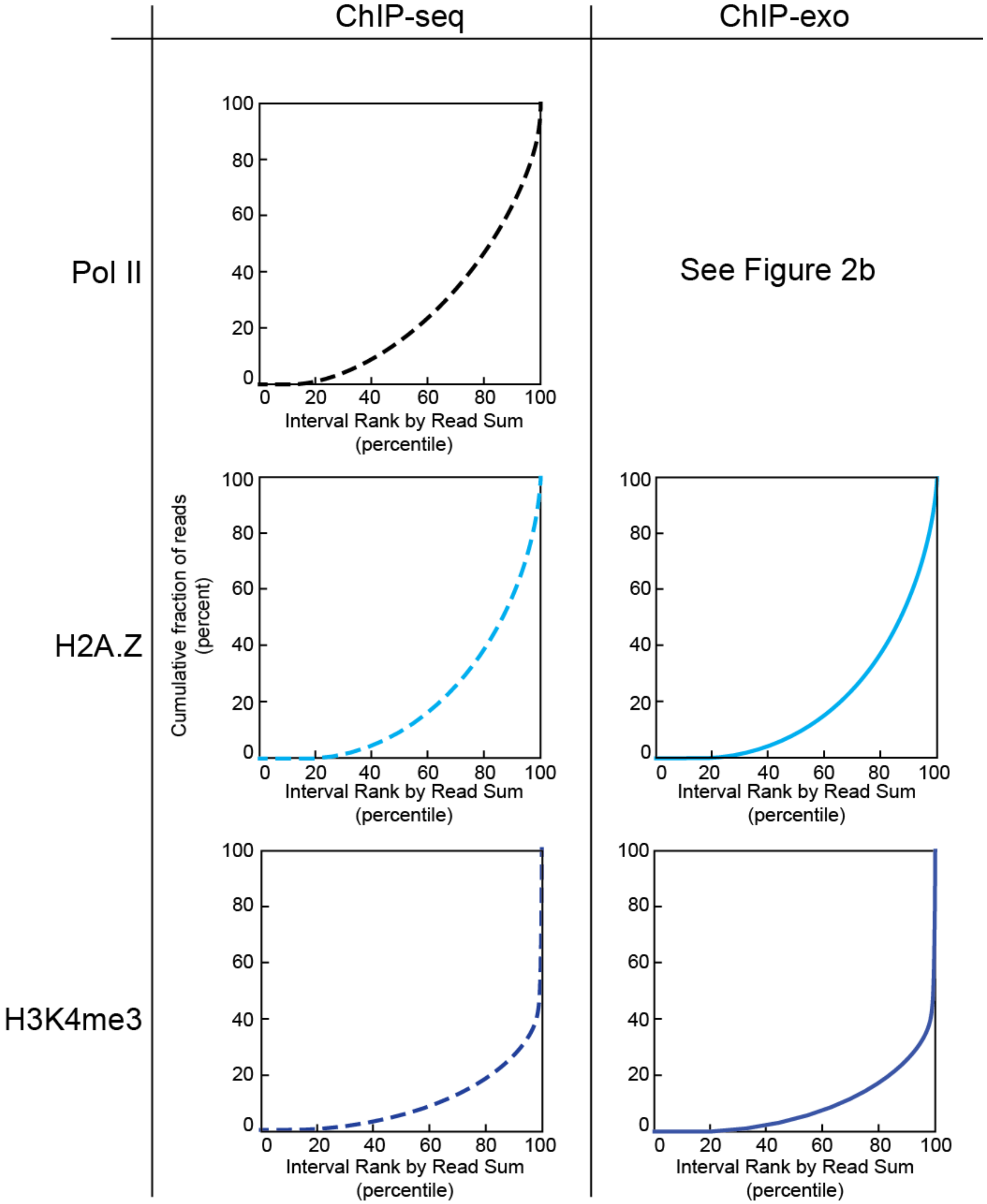

Supp. Fig. 3. Fingerprint plots to assess genome coverage and ChIP enrichment for ChIP-seq and ChIP-exo from merged biological replicates. Related to Fig. 2b.

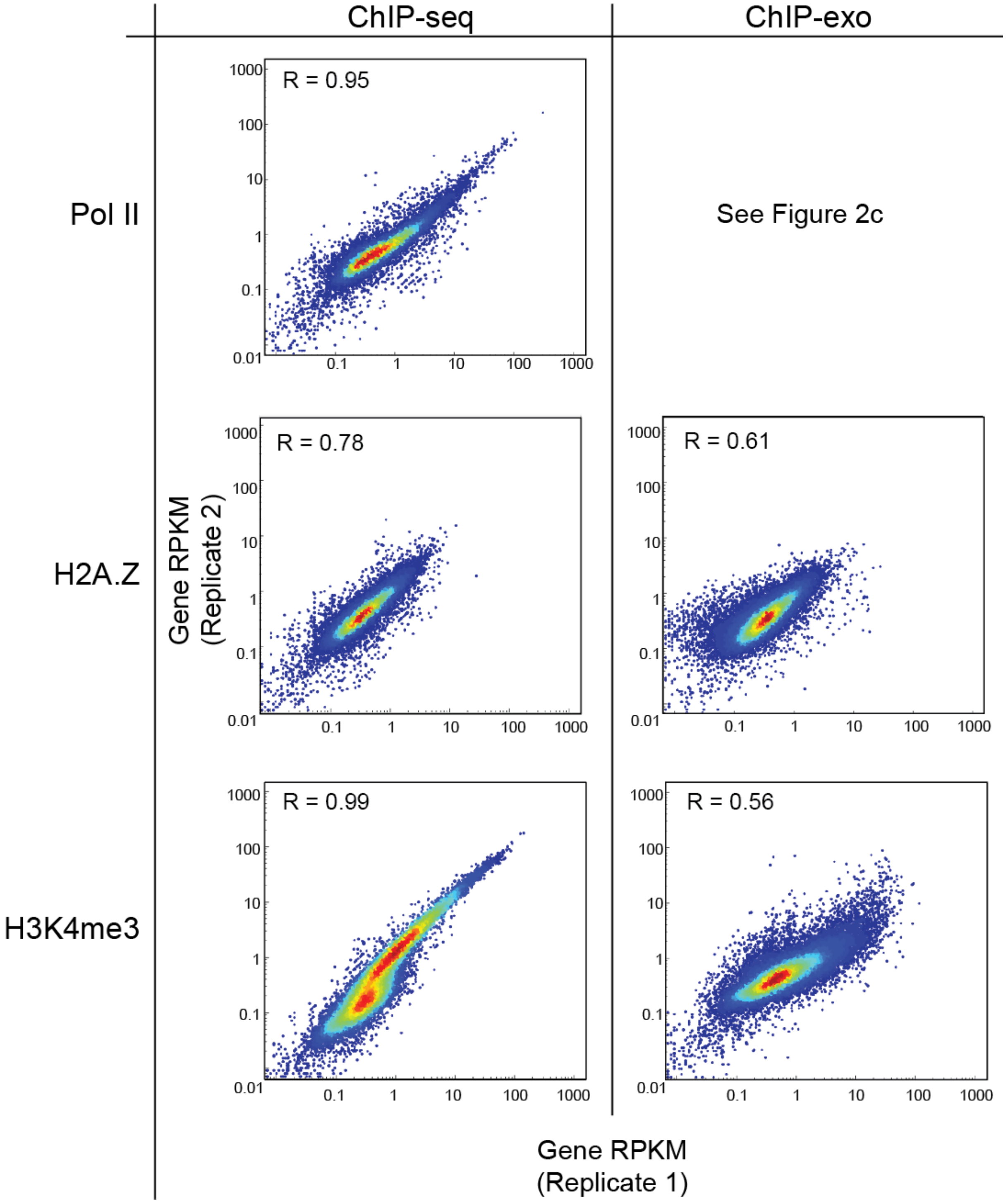

Supp. Fig. 4. Scatter plot correlation analysis for Pol II ChIP-exo biological replicates as measured by the Spearman correlation coefficient R-values (upper left corner). Related to Fig. 2c.

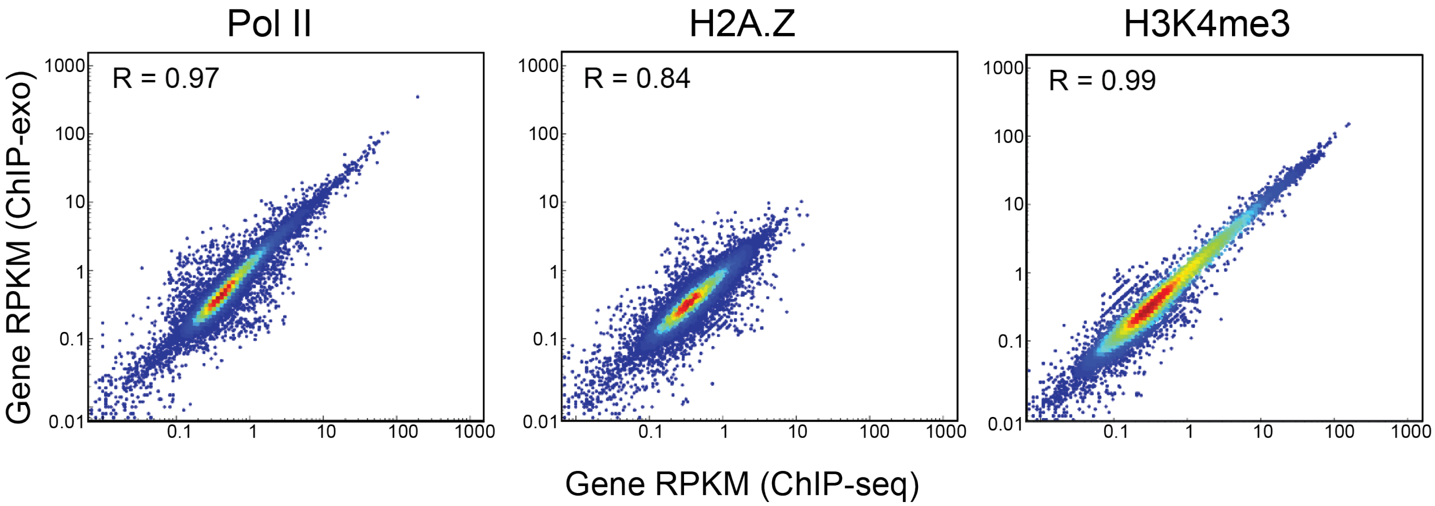

Supp. Fig. 5. Scatter plot correlation analysis for ChIP-exo and ChIP-seq libraries as measured by the Spearman correlation coefficient R-values (upper left corner). Related to Fig. 2c and Supp. Fig. 4.

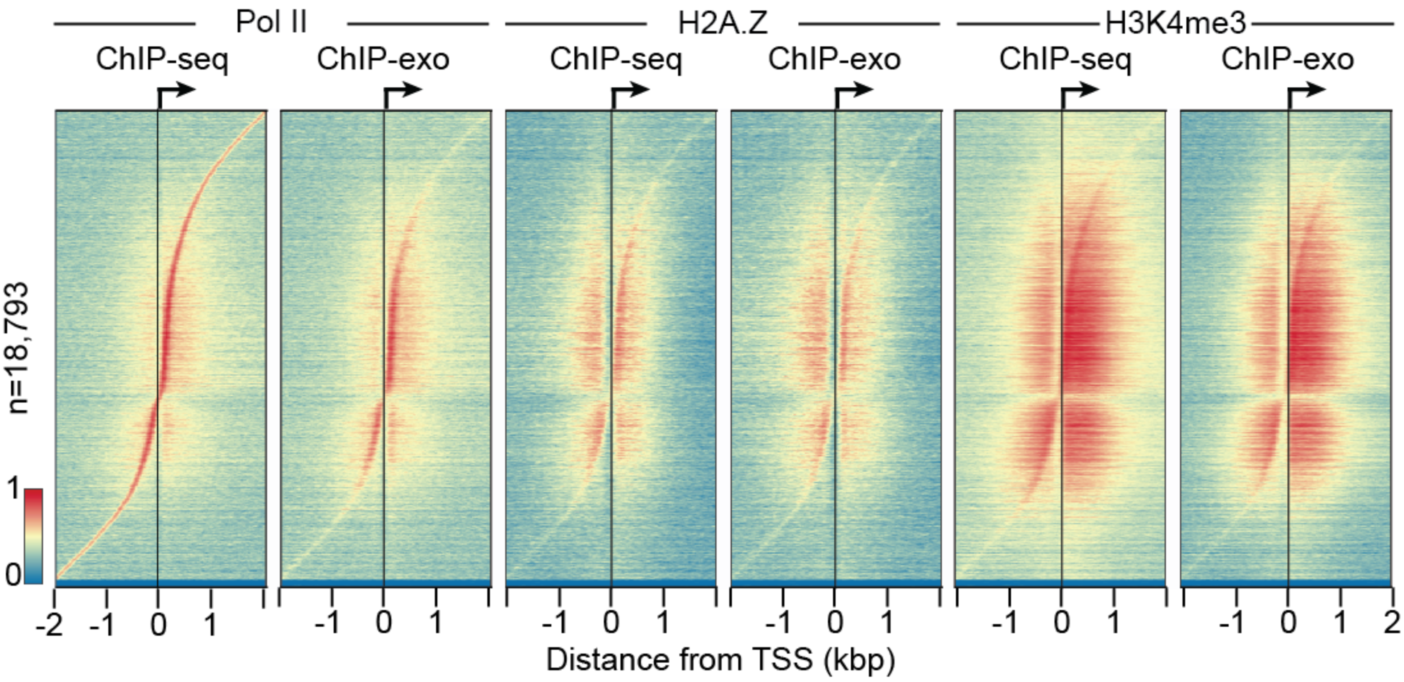

Supp. Fig. 6. ChAsE heatmap display for Pol II, H2A.Z, and H3K4me3 ChIP signal from merged biological replicates. Rows are linked and sorted by Pol II ChIP-seq max peak position. Related to Fig. 3a.
